# Brussels Chicory Enhances Exhaustive Aerobic Exercise Performance and Post-Exercise Recovery, Possibly Through Promotion of Lactate Oxidation: A Pilot Randomized, Single-Blind, Placebo-Controlled, Two-Way Crossover Study

**DOI:** 10.3390/nu17020365

**Published:** 2025-01-20

**Authors:** Yihui Mao, Junhao Huang, Shuangshuang Li, Guanyu Chen, Yushi Du, Mengxi Kang, Shasha Zhu, Wenyu Zhang, Qiuhui Xu, Yihan Wang, Wenhua Ling, Xijuan Luo, Dongliang Wang

**Affiliations:** 1 Department of Nutrition, School of Public Health, Sun Yat-sen University (Northern Campus), 74 Zhongshan Road II, Guangzhou 510080, China; maoyh23@mail2.sysu.edu.cn (Y.M.); lishsh86@mail2.sysu.edu.cn (S.L.); chengy268@mail2.sysu.edu.cn (G.C.); duysh5@mail2.sysu.edu.cn (Y.D.); kangmx@mail2.sysu.edu.cn (M.K.); zhushsh8@mail2.sysu.edu.cn (S.Z.); zhangwy69@mail2.sysu.edu.cn (W.Z.); xuqh36@mail2.sysu.edu.cn (Q.X.); wangyh266@mail2.sysu.edu.cn (Y.W.); lingwh@mail.sysu.edu.cn (W.L.); 2 Guangdong Provincial Key Laboratory of Sports and Health Promotion, Scientific Research Center, Guangzhou Sport University, Guangzhou 510500, China; junhaohuang2006@hotmail.com; 3 Guangdong Provincial Key Laboratory for Food, Nutrition and Health, Guangzhou 510080, China; 4 Department of Sports, Sun Yat-sen University, 135 West Xingang Road, Guangzhou 510275, China

**Keywords:** Brussels chicory, phenolic acid, exercise performance, LDHB, lactate metabolism

## Abstract

Background: Brussels chicory affluent in phenolic acids could inhibit atherosclerosis; however, its effects on exercise performance and post-exercise recovery are unknown. We hypothesized that Brussels chicory could enhance exhaustive aerobic exercise performance and post-exercise recovery by promoting lactate oxidation. Methods: This is a single-blind, randomized, placebo-controlled two-way cross-over trial involving 32 untrained college students (men 18) who consumed either Brussels chicory juice (100 g of Brussels chicory containing ~130 mg phenolic acids and 180 mL fresh milk) or placebo (180 mL fresh milk) for 7 days with a 2-week washout period. On the 7th day, participants received a short-term, progressive workload, high-intensity, exhaustive aerobic exercise with the Bruce protocol. Time to exhaustion and blood lactate were evaluated after exercise. C2C12 myotubes were treated with Brussels chicory phenolic acids (0.625–10 μM) to evaluate these effects on lactate metabolism and lactate dehydrogenase A (LDHA) and B (LDHB), two enzymes responsible for lactate biosynthesis and oxidation, respectively. Results: Brussels chicory consumption increased time to exhaustion by 8.3% and 12.2% for men and women participants, respectively. This administration also promoted post-exercise recovery, evidenced by a reduction in blood lactate (14.5% for men and 10.6% for women). In C2C12 myotubes, Brussels chicory protocatechuic acid and caffeic acid did not affect LHDA-mediated lactate production, whereas these compounds dose-dependently promoted LDHB-mediated lactate oxidation through an enrichment of mitochondria LDHB. Conclusions: Dietary supplementation with Brussels chicory may enhance short-term, progressive workload, high-intensity, exhaustive aerobic exercise performance and post-exercise recovery in humans, possibly by accelerating LDHB-mediated lactate oxidation.

## 1. Introduction

An optimal performance in exercise training/competition and post-exercise recovery is influenced by a dynamic interplay of various physiological, psychological, and environmental factors, among which environmental dietary practices stand as pivotal elements as ergogenic agents [1,2]. A relationship among the type, amount, and timing of dietary nutrients (e.g., carbohydrates and proteins) and optimal exercise performance is well established [3]. However, less is known about the role of dietary polyphenols that mainly consist of flavonoids and phenolic acids, non-nutrient components in plant foods, in achieving an optimal exercise performance.

It has long been thought that accumulation of lactate, an end-product of glycolysis enhanced by high-intensity short-term exercises [4], causes metabolic acidosis in the muscles, a phenomenon which may contribute to muscle fatigue and a delay in post-exercise recovery [5,6,7]. Different from the classical viewpoints on lactate, ample evidence from the 1980s has shown that disposal of lactate by either oxidation or gluconeogenesis, the two major fates of lactate, consumes protons and, in turn, elicits an alkalinizing effect in both muscle fibers and blood [8]. Brooks’ group have elegantly shown that lactate disposal is mainly through oxidation, especially during exercise, when oxidation accounts for 70–75% of removal, and gluconeogenesis eliminates the remainder [9]. This previously unrecognized role of lactate raises the possibility that lactate is an ergogenic agent in certain physical activities [10,11,12,13,14]. Different independent groups have reported that oral consumption of lactate could increase exercise performance during short, high-intensity work bouts to exhaustion in adults by 4–26% [10,11,12,13,14]. The action mechanism is partially due to the promotion of lactate oxidation and a concordant improvement in acid–base buffers [10,11,12,13,14]. However, the ergogenic effect of lactate consumption is non-conclusive, as there are also other human studies reporting no ergogenic effects [15,16]. Although a definite cause-and-effect relationship between lactate oxidation and an optimal performance in exercise training and post-exercise recovery in humans has not yet been established, the prospect of a role for lactate oxidation in achieving an optimal exercise performance remains enticing.

Lactate accumulation is determined by an imbalance between its production and its clearance [8]. On the one hand, glucose in the cytosol of muscle fibers is converted into pyruvate through a series of glycolysis reactions. When pyruvate production exceeds its oxidation in the mitochondria of muscle fibers, pyruvate is directly reduced to lactate, a process dependent on cytosol lactate dehydrogenase A (LDHA) [4]. On the other hand, the clearance of lactate mainly depends on the muscle fibers’ aerobic metabolic capacity. Lactate is converted into pyruvate under the action of mitochondria lactate dehydrogenase (LDHB) that then enters the Krebs cycle to yield ATP [17]. The cluster of differentiation 147 (CD147), a transmembrane glycoprotein in the mitochondria, is required for mitochondria LDHB to convert lactate into pyruvate [18].

Dietary polyphenols are widely found in grains, cereals, pulses, vegetables, fruits, spices, chocolates, tea, coffee, and wine [19]. Copious epidemiolocal and interventional studies have shown that the consumption of polyphenols holds promise to offer protection against various ailments in humans, such as cardiovascular diseases, type 2 diabetes, metabolic syndrome, and non-alcoholic fatty liver disease [20]. However, the human exercise data are somewhat limited in comparison to the health-promoting studies. To date, a variety of human studies has shown that flavonoid-rich foods, such as tart cheery, pomegranate, coffeeberry, and phenolic-acid-rich coffee, possess an ergogenic activity evidenced by an enhancement of exercise performance and post-exercise recovery, though other studies report no ergogenic effects [21,22,23]. These action mechanisms remain largely unknown; however, anti-inflammation, antioxidation, improvement of glucose and fatty acid oxidation, modulation of lactate metabolism, and/or improvement of blood flow have been proposed for these ergogenic activities [24,25].

Brussels chicory (Asteraceae, *Cichorium intybus* L. *var. foliosum*; also called French or Belgian endive), a typical leafy vegetable in the Mediterranean diet, is rich in phenolic acids including protocatechuic acid, gallic acid, p-hydroxybenzoic acid, chlorogenic acid, caftaric acid, and caffeic acid, in addition to some flavonoids and terpenes in smaller quantities [26,27]. Our series of preclinical studies have shown that Brussels chicory could inhibit atherosclerosis development with the help of its phenolic acid constitute [28,29,30,31]; however, its effect on human exercise is lacking. Indeed, phenolic acids have been documented to regulate lipid, glucose, and cellular energy metabolism in vivo, apart from these well-known antioxidant and anti-inflammatory properties [32,33,34,35,36]. In the present study, we hypothesize that daily intake of phenolic-acid-rich Brussels chicory could enhance exercise performance and post-exercise recovery by regulating lactate metabolism using the Bruce protocol, a well-known high-intensity, exhaustive aerobic exercise characterized by high glycolytic fluxes and a metabolic acidosis.

## 2. Material and Methods

### 2.1. Study Participants

This randomized, placebo-controlled cross-over trial was conducted from July to October 2023 in Guangzhou, China, with participants recruited from local universities. Inclusion criteria were healthy, recreationally active college students (ages 18–45) with a BMI of 18.5–23.9 kg/m^2^, moderate physical activity levels (600–3000 MET-min/week), and no significant health issues based on medical history and body composition measurements. Participants agreed to maintain their current diet and physical activity levels and to comply with the study procedures. Exclusion criteria included metabolic, infectious, serious heart/kidney diseases, drugs affecting nutrient metabolism, smoking, or alcohol intake over 15 g/day.

### 2.2. Randomization and Blinding

Participants were randomly assigned to either the Brussels chicory or the placebo group using a computer-generated randomization process, stratified by gender. A total of 100 serial numbers were allocated equally (1:1) to the two groups using block randomization with a block size of four. These serial numbers were printed and affixed to opaque boxes containing either the Brussels chicory or placebo, according to group allocation. Randomization was overseen by an independent researcher who was not involved in recruitment, data collection, laboratory analyses, or data interpretation. The allocation was blinded to both investigators and data analysts.

### 2.3. Study Design and Trial Protocol

This cross-over study included a 7-day intervention with at least a 2-week washout period in between. The Brussels chicory group received a home-made Brussels chicory juice containing 100 g of Brussels chicory (JIAYAONONGYE, Shijiazhuang, China) mixed with 180 mL of fresh milk (SHINY MEADOW, Inner Mongolia Mengniu Dairy (Group) Limited by Share Ltd., Hohhot, China) every day, while the placebo group received 180 mL of fresh milk. A total of 100 g of Brussels chicory contains 1.3 g of proteins, 0.2 g of fat, 3.3 g of carbohydrates, 1.2 g of crude fiber, and 93.8 g of moisture [37]. Participants were instructed to maintain their usual diet and physical activity throughout the study and to abstain from exercise as well as from consuming coffee, tea, energy drinks, or alcohol in the 48 h before the exhaustive exercise aerobic test.

On the 7th day during the intervention, participants consumed a standardized breakfast (a McDonald’s burger without vegetables and fruit, providing 498 kcal and 24 g of fat with 42.1% of energy) within 10 min, along with the assigned Brussels chicory juice or placebo. A total of 90 min after eating, they performed an exhaustive exercise test following the Bruce protocol. After a two-week washout period, participants were crossed over to the alternate supplementation group for the second investigation phase.

### 2.4. Outcome Measurement and Sample Size Calculation

The primary outcome was the time to exhaustion during the exhaustive aerobic exercise. The secondary outcomes that consisted of metabolic and physiological responses were assessed at various time points. Maximal oxygen consumption (VO_2_max) was measured during the exhaustive aerobic exercise. Heart rate, systolic blood pressure (SBP), and diastolic blood pressure (DBP) were monitored 0, 5, 15, and 60 min after the exhaustive aerobic exercise using an electronic arm sphygmomanometer (Yuyue, Danyang, China). Blood samples were collected in EDTA plasma and serum vacutainer tubes 0 and 60 min after the exhaustive aerobic exercise. Plasma samples were immediately centrifuged, while serum samples were incubated at room temperature for 30 min before centrifugation at 4000× *g* for 20 min at 4 °C. Aliquots of plasma and serum were then stored at −80 °C for subsequent biochemical assays. Serum interleukin 6 (IL-6), IL-8, and C-reactive protein (CRP) were measured using human ELISA kits from Abcam (Cambridge, UK). Plasma superoxide dismutase (SOD), glutathione peroxidase (GSH-PX), and catalase (CAT) activity were measured using colorimetric kits from Abcam (Cambridge, UK). Blood pH and bicarbonate (HCO_3_^−^) were determined using an Abbott i-STAT 1 portable clinical analyzer (Abbott Laboratories, Chicago, IL, USA), calibrated as per the manufacturer’s guidelines. Small fingertip blood samples were collected 0, 5, 15, and 60 min after the exhaustive aerobic exercise for blood lactate and blood glucose measurements, using a portable lactate meter (ZHIXIANG, Guangzhou, China) and a blood glucose meter 560 (Yuyue, Danyang City, China), respectively.

Based on a previous study reporting a 6.5% mean difference in time to exhaustion with a standard deviation of 43.27 [38], the sample size was calculated using a two-tailed α level of 0.05 and an 80% power. The sample size calculation indicated that 14 participants were required per group. To account for a 10% dropout rate, 15 participants were included in each group.

### 2.5. Sociodemographic Data Collection and Anthropometric Assessments

Demographic data were collected at baseline using a structured questionnaire, which included information on gender, race, date of birth, education level, smoking status, alcohol consumption, dietary habits, physical activity levels, use of nutritional supplements, and medical history. At baseline, trained researchers measured height, weight, percentage of body fat, blood pressure, and heart rate using calibrated equipment and standardized procedures. The body mass index (BMI) was calculated as weight/height squared (kg/m^2^). Dietary intake and the physical activity level over the preceding week were assessed using a three-day 24 h dietary recall and a physical activity questionnaire [39], respectively. Dietary energy and nutrient intake were estimated based on the 2009 China Food Composition Database [40].

### 2.6. High-Intensity Exhaustive Aerobic Exercise from the Bruce Protocol

The exhaustive aerobic exercise test was conducted using the Bruce protocol on a calibrated treadmill (MasterScreen CPX, CareFusion, Butzbach, Germany) [41]. Before the test, the rate of perceived exertion (RPE) scale was thoroughly explained to the participants, who were informed that they would need to report their RPE midway through each 3 min stage. Participants practiced walking and running on the treadmill at various speeds until both the participants and the researchers were confident in their ability to safely perform the test. During the warm-up, participants walked on a flat treadmill at a speed of 1.7 mph for 3 min, then continued for another 3 min at the same speed with a 5% incline. This was subsequently followed by the continuous, incremental Bruce protocol. During the first stage, participants walked at 1.7 mph (2.7 km/h) with a 10% grade, while, in stage 2, the speed increased to 2.5 mph (4.0 km/h) with a 12% grade. Subsequent stages involved further increases in speed and grade at 3 min intervals until the participant reached volitional exhaustion (Table 1). Participants switched from walking to running when the demand became greater and were verbally encouraged to continue until volitional exhaustion (RPE: ≥18). The test was terminated when the participant could no longer proceed and grasped the treadmill handrail. The test was considered exhaustive if at least two of the following three criteria were met: a respiratory exchange ratio (RER) ≥ 1.10, a plateau in VO_2_ despite an increasing workload, or a heart rate of or above the age-predicted maximum [42]. Time to exhaustion was recorded, and VO_2_max was measured using an ergospirometric device with a breath-by-breath gas analysis system.

### 2.7. Phenolic Acids in Brussels Chicory and Serum Samples

Phenolic acids in Brussels chicory and serum samples were measured using high-performance liquid chromatography coupled with electrochemical detection (Agilent, Santa Clara, CA, USA), as we previously described [43]. Quantification was performed using an internal standard method. For each analytical run, a calibration curve was prepared in the appropriate matrix (methanol or blank serum) to ensure an accurate determination of phenolic acid concentrations in both the Brussels chicory and the serum samples.

### 2.8. Cell Culture

C2C12 myoblasts were sourced from the Cell Bank Institute at the Chinese Academy of Sciences (ICell Bioscience Inc., Shanghai, China) and maintained in a proliferation medium (high-glucose DMEM, Gibco, Grand Island, NY, USA) containing 10% fetal bovine serum (FBS, ThermoFisher, Waltham, MA, USA) in a humidified atmosphere with 5% CO_2_ at 37 °C. When the cells reached a 90% confluence, the medium was replaced with a differentiation medium (containing 2% horse serum, ThermoFisher, Waltham, MA, USA) to induce differentiation for 7 days into myotubes.

### 2.9. Cell Viability

Cell viability was measured by both methyl thiazolyl tetrazolium (MTT) and LDH release assays. The MTT assay was performed as we previously described [28]. The release of LDH was detected in cell culture supernatants using an in vitro toxicity assay kit (Sigma-Aldrich, St. Louis, MO, USA) as per the manufacturer’s protocols.

### 2.10. Measurements of Intracellular and Conditioned Medium Lactate Concentrations

After the C2C12 myotubes were treated with Brussels chicory’s phenolic acids (Yuanye, Shanghai, China), including protocatechuic acid, gallic acid, p-hydroxybenzoic acid, chlorogenic acid, caftaric acid, and caffeic acid, for 24 h, 20 mM lactate was added to the medium. The intracellular and conditioned medium lactate concentrations were measured after the C2C12 myotubes were incubated for 6 h. Following aspiration of all spent medium, the C2C12 myotubes were washed three times with cold PBS, 300 uL of cold PBS was added to the plate, and the C2C12 myotubes were scraped and frozen at −80 °C until further processing for the measurement of intracellular lactate. To ensure proper cell disruption, the cell suspensions were sonicated for 8 s at a 50% amplitude using a pencil-type sonicator (Sonics, Newtown, CT, USA). Protein concentrations were determined using the Quick Start^TM^ Bradford Protein Assay kit 1 (Bio-Rad, Hercules, CA, USA) as per manufacturer’s protocol. The supernatant was collected after centrifugation at 14,000× *g* for 15 min. Lactate concentrations in both the conditioned medium and intracellular (lysed cell supernatant) samples were measured using an L-lactate assay kit (Eton Biosciences, San Diego, CA, USA) according to the manufacturer’s protocol. Protein and lactate concentrations were determined via colorimetry using a BioTek 800 TS absorbance reader (Agilent, Santa Clara, CA, USA). Intracellular and conditioned medium lactate concentrations were normalized to the respective protein concentrations prior to further calculations/data analysis.

### 2.11. Seahorse Oxidative and Glycolytic Metabolic Assay

The Seahorse XFe24 Extracellular Flux Analyzer equipped with Seahorse XF24 FluxPaks from Agilent Technologies (Santa Clara, CA, USA) was utilized to determine the oxygen consumption rate (OCR) and extracellular acidification rate (ECAR) of 1 × 10^3^ viable C2C12 myoblasts in each well. In the OCR measurements, the initial OCR (basal OCR) in pmol/min was recorded, followed by measurements post-injection of 2 µM oligomycin (an ATP synthase inhibitor), 0.5 µM carbonyl cyanide-p-trifluoromethoxy phenylhydrazone (CCP, a protonophore that uncouples oxidative phosphorylation), 0.5 µM rotenone (an inhibitor of complex I), and 0.5 µM antimycin A (an inhibitor of complex III). Regarding ECAR, the C2C12 myoblasts were seeded in an XF base medium (Agilent Technologies, Santa Clara, CA, USA), supplemented with 200 mM L-glutamine and 5 mM 2-[4-(2-hydroxyethyl) piperazin-1-yl] ethanesulfonic acid (HEPES), following the manufacturer’s recommendations for glycolytic assays. Prior to use, the sensor cartridge was prepared by adding 1 mL of XF calibrant to each well and incubating at 37 °C for one day. The sensor cartridge’s injection ports were sequentially filled with 10 mM glucose, 2 µM oligomycin, 0.5 µM rotenone, a combined injection of 0.5 µM antimycin A, and 50 mM 2-deoxyglucose, corresponding to four timed injections designed to achieve the specified final concentrations within the wells.

### 2.12. Isolation of Mitochondria

Cell collection was performed using a mitochondrial isolation buffer composed of 210 mM mannitol, 60 mM sucrose, 10 mM KCl, 10 mM sodium succinate, 5 mM EGTA, 2 mM HEPES-KOH (pH 7.4), 0.5 mM DTT, 1 mM PMSF, and the COMPLETE protease inhibitor cocktail (Merck, Kenilworth, NJ, USA). The mitochondria were isolated by differential centrifugation as described earlier [44]. The resulting mitochondrial pellet was tested for the presence of mitochondrial and endoplasmic reticulum marker enzymes. The cytosolic fraction was separated by centrifuging the supernatant from the post-mitochondrial spin at 120,000× *g* for 1 h using an Optima XE-100 microcentrifuge (Beckman coulter, Miami, FL, USA).

### 2.13. Western Blot

Protein samples were separated by SDS-PAGE. The method for western blotting has been previously described [28]. Primary antibodies included LDHB (#A18096, Abclonal, Wuhan, China), LDHA (#2012, Cell Signaling, Danvers, MA, USA), CD147 (#230921, Abcam, Cambridge, UK), GADPH (#60004-1-Ig, Proteintech, Wuhan, China), and VDAC1 (#ab235143, Abcam, Cambridge, UK).

### 2.14. RNA Preparation and Quantitative Real-Time PCR (qRT-PCR) Analysis

Total RNA was extracted from cells using RNeasy kits, followed by reverse transcription with the SuperScript III kit x (BioRad, Hercules, CA, USA) using random primers. The mRNA levels of LDHB, LDHA, CD147, and GAPDH were quantified by qRT-PCR using the iQ SYBR Green Supermix. The primer sequences for all the genes examined in this study were from the PrimerBank website (http://pga.mgh.harvard.edu/primerbank/), accessed on 1 April 2024. GAPDH mRNA was used as an internal control for normalization, and mRNA expression levels were quantified using the 2^(−ΔΔCt)^ method.

### 2.15. Small Interfering RNA (siRNA) Strategy

C2C12 myotubes were transfected with siRNA directed against LDHB (*siLDHB*), CD147 (*siCD147*), or nontargeting siRNA (siCtr) with the use of the Lipofectamine RNAiMAX reagent (Thermo Fisher Scientific, Waltham, MA, USA) according to the manufacturer’s protocol. The knockdown efficiency of LDHB and CD147 was assessed by western blot analysis.

### 2.16. Lactate Oxidation Assay

C2C12 myoblasts, at a density of 7000 cells per well, were grown in 96-well CellBIND^®^ microplates. After a 24 h preincubation with Brussels chicory’s phenolic acids, the cells were treated with L-[^14^C(U)] lactate at a concentration of 1 µCi/mL for a period of 6 h to allow for CO_2_ capture, as previously detailed [45]. A 96-well UniFilter^®^ microplate, preconditioned with a soak in 1M NaOH, was placed atop the CellBIND^®^ plate to trap the produced CO_2_ during the 6 h incubation at 37 °C. The emission of CO_2_ and the radioactivity associated with the cells (CA) were quantified utilizing a PerkinElmer MicroBeta scintillation counter (PerkinElmer, Waltham, MA, USA). The extent of complete oxidation was calculated as CO_2_/(CO_2_ + CA), which was normalized to the cellular protein content.

### 2.17. Lactate Dehydrogenase Activity

LDHB activity was assayed in isolated mitochondria using a commercial lactate dehydrogenase activity assay kit (Sigma-Aldrich, MO, USA), according to the manufacturer’s instructions.

### 2.18. Statistical Analyses

In this cross-over study, all statistical analyses adhered to the intention-to-treat (ITT) principle. Continuous variables were expressed as mean ± SD or median (IQR), while categorical variables were presented as percentages (%). Independent Student’s *t*-tests, Mann–Whitney U, and chi-square tests were used to compare continuous and categorical variables, respectively. The area under the concentration–time curve (AUC) for blood lactate, glucose, heart rate, SBP, and DBP was calculated using the DAS 2.1 software (BioGuider Co., Changzhou, China). A one-way repeated measures ANOVA was used to compare time to exhaustion between the Brussels chicory and the placebo groups. Secondary outcomes, including serum phenolic acid levels, VO_2_max, blood bicarbonate, pH, and AUC were analyzed using one-way repeated measures ANOVAs. Blood lactate, blood glucose, heart rate, SBP, DBP, inflammation, and oxidative stress markers were assessed using two-way repeated measures ANOVAs with the Bonferroni correction (*p* < 0.008). For in vitro experiments, data were analyzed with independent Student’s *t*-tests or ANOVAs. A *p*-value < 0.05 was considered statistically significant. Analyses were performed using SPSS version 26.0 (SPSS Inc., Chicago, IL, USA). NS indicates nonsignificant.

## 3. Results

### 3.1. Participants’Characteristics

**Figure 1:**
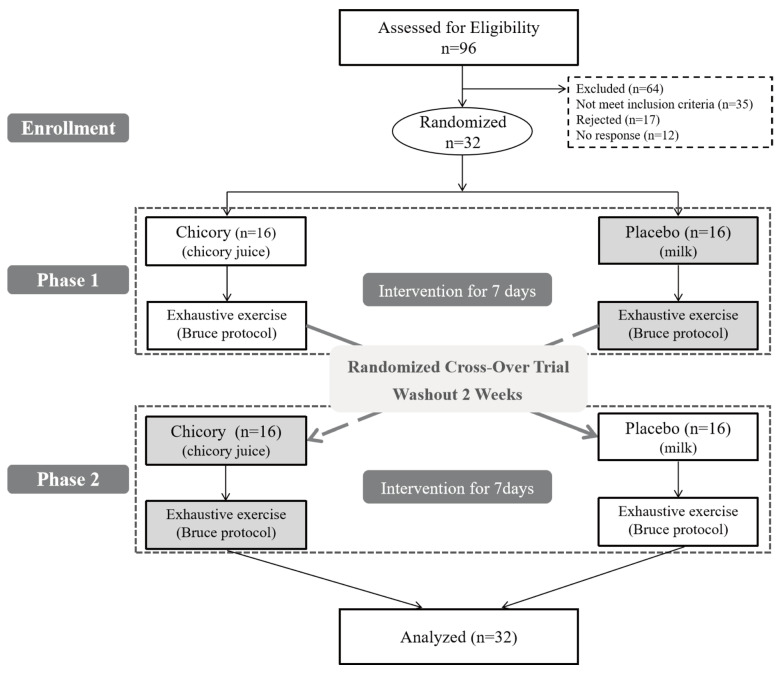
CONSORT (Consolidated Standards of Reporting Trials) flow diagram indicating the design of the trial.

A total of 96 healthy individuals aged between 18 and 28 years were screened for this study, with 32 meeting the inclusion and exclusion criteria and being subsequently enrolled. No participants withdrew or dropped out during the study (Figure 1). These participants were randomly assigned to either the Brussels chicory group or the placebo group. Baseline characteristics, including weight, BMI, percentage of body fat, SBP, DBP, and heart rate, were comparable between the Brussels chicory and the placebo groups (all *p* > 0.05). Energy and macronutrient intake (carbohydrates, proteins, and fat) and physical activity level and type were also similar (all *p* > 0.05) (Table 2).

### 3.2. Brussels Chicory Consumption Increased Time to Exhaustion During Exhaustive Aerobic Exercise in Healthy Participants

**Figure 2:**
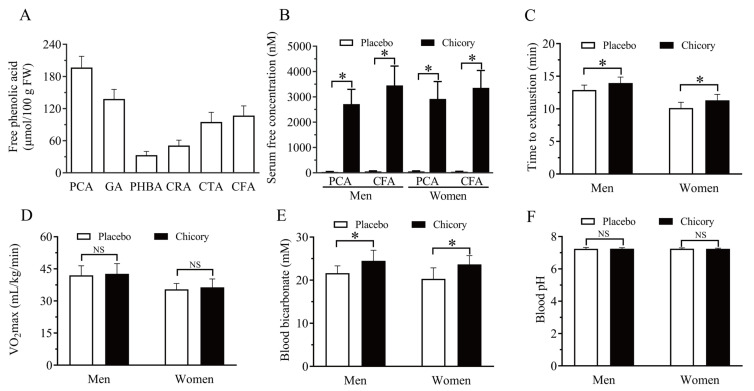
Effect of Brussels chicory on exercise performance. Content of the free phenolic acids in Brussels chicory (*n* = 3) (**A**), serum concentrations of protocatechuic acid and caffeic acid 0 min after exhaustive aerobic exercise (**B**), effect of dietary supplementation with Brussels chicory on time to exhaustion (**C**) and VO_2_max (**D**) in exhaustive aerobic exercise, blood bicarbonate (**E**) and pH (**F**) levels 0 min after exhaustive aerobic exercise. Data are mean ± SD. (**B**–**F**), *n* = 32. One-way repeated measures ANOVA, * *p* < 0.05, significantly different from placebo. Abbreviation: VO_2_max, maximal oxygen consumption; PCA, protocatechuic acid; GA, gallic acid; PHBA, p-hydroxybenzoic acid; CRA, chlorogenic acid; CTA, caftaric acid; CFA, caffeic acid; ANOVA, analysis of variance.

The phenolic acids in Brussels chicory include hydroxybenzoic acids such as protocatechuic acid (196.7 ± 21.2 µmol/100 g FW), gallic acid (138.1 ± 18.5 µmol/100 g FW), and p-hydroxybenzoic acid (33.5 ± 7.3 µmol/100 g FW), as well as hydroxycinnamic acids including chlorogenic acid (51.6 ± 8.1 µmol/100 g FW), caftaric acid (95.4 ± 16.5 µmol/100 g FW), and caffeic acid (107.3 ± 15.4 µmol/100 g FW) (Figure 2A). In the Brussels chicory group, free protocatechuic acid and caffeic acid levels in serum were 2712.7 ± 583.4 nM and 3452.4 ± 767.8 nM, respectively, for men, and 2918.5 ± 689.3 nM and 3358.9 ± 687.8 nM, respectively, for women 0 min post-exercise. In the placebo group, these levels were close to zero (Figure 2B). Additionally, time to exhaustion was significantly longer in the Brussels chicory group compared to the placebo group, with an increase of 8.3% in men (*p* = 0.001) and 12.2% in women (*p* < 0.001) (Figure 2C). However, no significant differences were observed in VO_2_max between the Brussels chicory and the placebo groups for both men (*p* = 0.65) and women (*p* = 0.61) (Figure 2D). Notably, blood HCO_3_^−^ levels were significantly higher in the Brussels chicory group, with increases of 13.2% for men (*p* = 0.001) and 16.6% for women (*p* < 0.001) (Figure 2E). However, no significant differences were found in blood pH levels between the Brussels chicory and the placebo groups for both men (*p* = 0.48) and women (*p* = 0.39) (Figure 2F).

### 3.3. Brussels Chicory Consumption Promoted Post-Exercise Recovery in Healthy Participants

**Figure 3:**
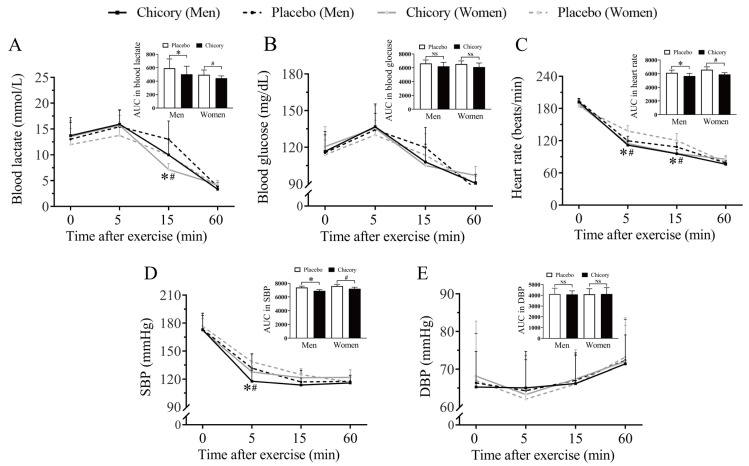
Effect of Brussels chicory on post-exercise recovery. The concentrations and areas under the curve (AUC) of blood lactate (**A**), blood glucose (**B**), heart rate (**C**), SBP (**D**), and DBP (**E**) 0, 5, 15, and 60 min after exhaustive aerobic exercise. Data are mean ± SD (*n* = 32). *p* < 0.05 when compared by two-way repeated measures ANOVA followed by the Bonferroni correction for post-hoc comparisons. Differences in AUC between the Brussels chicory and the placebo groups were analyzed using a one-way repeated measures ANOVA. * *p* < 0.05, significantly different from placebo in men. ^#^ *p* < 0.05, significantly different from placebo in women, NS, nonsignificant. Abbreviation: AUC, area under the curve; SBP, systolic blood pressure; DBP, diastolic blood pressure; ANOVA, analysis of variance.

In both men and women, the blood lactate concentration patterns in the Brussels chicory group and the placebo group both showed a significant time effect (all *p* < 0.001). Blood lactate curves showed significant differences 15 min post-exercise, with lower levels in the Brussels chicory group compared to the placebo group: a reduction of 23.2% in men (*p* = 0.001) and 28.0% in women (*p* < 0.001). The area under the blood lactate curve was also significantly lower for the Brussels chicory group: a reduction of 14.5% in men (*p* = 0.001) and 10.6% in women (*p* = 0.021) (Figure 3A). In both men and women, the blood glucose concentration patterns in the Brussels chicory and placebo groups exhibited a significant time effect (all *p* < 0.05). Although blood glucose levels showed a decline 15 min post-exercise, no significant differences were observed between the Brussels chicory and the placebo groups (Figure 3B). Moreover, in both men and women, the heart rate patterns in the Brussels chicory and the placebo groups showed a significant time effect (all *p* < 0.05), with the Brussels chicory group exhibiting lower values 5 and 15 min post-exercise compared to the placebo group. (Figure 3C). SBP was also significantly lower in the Brussels chicory group 5 min post-exercise in men and women (Figure 3D), but there were no significant differences in DBP (Figure 3E). Regarding inflammation and oxidative stress markers, there were no significant differences in IL-6, IL-8, CRP, SOD activity, GSH-PX activity, and CAT activity between the Brussels chicory and the placebo groups 0 and 60 min after exhaustive aerobic exercise, as shown in Table 3 and Table 4.

### 3.4. Brussels Chicory Phenolic Acids Improved Lactate Metabolism in C2C12 Myotubes

**Figure 4:**
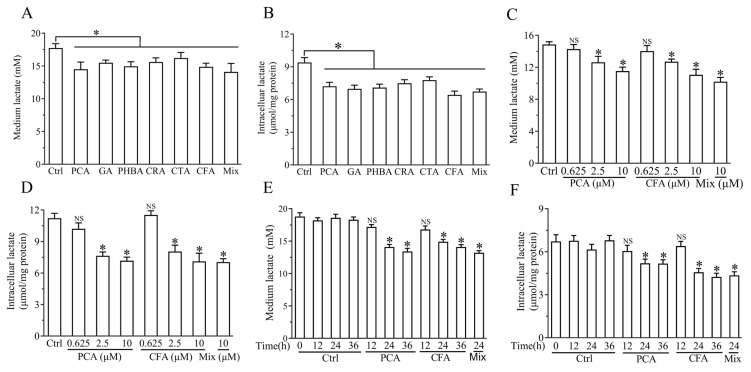
Effect of Brussels chicory phenolic acids on the levels of intracellular and conditioned medium lactate in myotubes. C2C12 myotubes were exposed to 10 μM of seven Brussels chicory phenolic acids, including PCA, GA, PHBA, CRA, CTA, and CFA, either individually or as a mix, for 24 h. After a 6 h incubation with 20 mM lactate, intracellular and conditioned medium lactate concentrations were measured (**A**,**B**). To further explore the effects, C2C12 myotubes were treated with PCA and CFA at multiple concentrations, as well as with a 10 μM mix for 24 h (**C**,**D**) or were exposed to a 10 μM of PCA and CFA at different time intervals (**E**,**F**). Data are mean ± SEM (*n* = 6), Student’s *t* test. (**A**–**D**), * *p* < 0.05 vs. control. (**E**,**F**), * *p* < 0.05 vs. control at 12, 24, and 36 h, respectively. NS, nonsignificant. Abbreviation: PCA, protocatechuic acid; GA, gallic acid; PHBA, p-hydroxybenzoic acid; CRA, chlorogenic acid; CTA, caftaric acid; CFA, caffeic acid; Mix, containing equal molar concentrations of all tested Brussels chicory phenolic acids.

To assess the role of Brussels chicory phenolic acids in lactate metabolism, C2C12 myotubes were incubated with 10 μM of seven Brussels chicory phenolic acids, including three benzoic acids (protocatechuic acid, gallic acid, and p-hydroxybenzoic acid), three cinnamic acid (chlorogenic acid, caftaric acid, and caffeic acid), or a 10 μM mixture (Mix) containing equal molar concentration of all six Brussels chicory phenolic acids for 24 h, followed by the addition of 20 mM lactate to the medium for 6 h. As shown in Figure 4A,B, the treatment with Brussels chicory phenolic acids reduced both intracellular and conditioned medium lactate levels compared to control. Given the comparable effects of these phenolic acids in the modulation of lactate levels in intracellular and conditioned medium and Brussels chicory rich in these phenolic acids, we chose protocatechuic acid, caffeic acid, and the mix with benzoic acid and cinnamic acid as representatives for benzoic acid, cinnamic acid, and Brussels chicory phenolic acids, respectively. The dose–effect assay showed that 0.625 μM of Brussels chicory protocatechuic acid and caffeic acid did not significantly reduce intracellular and conditioned medium lactate levels. Conversely, when the dosages of Brussels chicory protocatechuic acid and caffeic acid were increased to 2.5 and 10 μM, these compounds significantly reduced both intracellular and conditioned medium lactate levels (Figure 4C,D). In addition, time–effect assays further showed that both Brussels chicory protocatechuic acid and caffeic acid reduced the intracellular and conditioned medium lactate levels compared to the control (Figure 4E,F). The effects of Brussels chicory phenolic acids on lactate metabolism were associated with nonsignificant changes in the viability of C2C12 myotubes (evaluated by the MTT and LDH leakage assay) (Figure S1).

### 3.5. Brussels Chicory Phenolic Acids Promoted Lactate Clearance by Targeting LDHB-Mediated Lactate Oxidation

**Figure 5:**
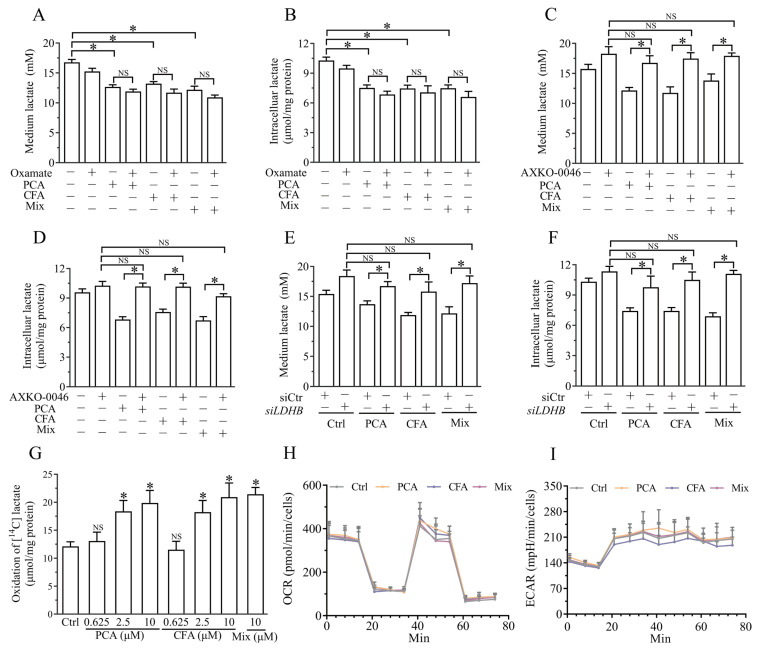
Brussels chicory’s phenolic acids promote lactate oxidation via targeting LDHB in myotubes. C2C12 myotubes were pre-treated with 10 μM PCA, CFA, or a mix for 24 h, then incubated with lactate for another 6 h in the presence or absence of AXKO-0046 (an inhibitor of lactate oxidation) or oxamate (an inhibitor of lactate production). Intracellular and conditioned medium lactate levels were measured in C2C12 myotubes treated with AXKO-0046 (**A**,**B**) and oxamate (**C**,**D**). C2C12 myotubes with LDHB knockdown were treated with PCA, CFA, or the mix for 24 h, followed by a 6 h incubation with lactate. Intracellular and conditioned medium lactate levels were then measured (**E**,**F**). C2C12 myotubes pre-treated with PCA, CFA, and the mix were incubated with [^14^C] lactate for 6 h to measure oxidation to CO_2_, which was trapped and quantified by liquid scintillation (**G**). Mitochondrial function was assessed using the Seahorse XFe96 analyzer, measuring OCR (**H**) and ECAR (**I**). Data are mean ± SEM (*n* = 6), Student’s *t* test, * *p* < 0.05 vs. control for PCA, CFA, or the mix. (**H**,**I**) Data are mean ± SEM (*n* = 6), two-way repeated measure ANOVA. NS, nonsignificant. Abbreviation: LDHB, lactate dehydrogenase B; PCA, protocatechuic acid; CFA, caffeic acid; Mix, containing equal molar concentrations of all six Brussels chicory phenolic acids; OCR, oxygen consumption rate; ECAR, extracellular acidification rate.

Lactate homeostasis is regulated by its oxidation clearance and synthesis. On the one hand, the effect of Brussels chicory protocatechuic acid, caffeic acid, and mix on promoting lactate clearance in C2C12 myotubes was abolished by treatment with AXKO-0046, an LDHB (a limiting enzyme in lactate oxidation) inhibitor (Figure 5A,B). In contrast, oxamate, an LDHA (a limiting enzyme in lactate synthesis) inhibitor, did not significantly affect the effect of Brussels chicory protocatechuic acid, caffeic acid, and mix on lactate metabolism (Figure 5C,D). Moreover, we knocked down LDHB through a small interfering RNA strategy. As shown in Figure S2, small interfering RNA against *LDHB* greatly inhibited its expression at the protein level. LDHB knockdowns almost negated the lactate clearance effects of Brussels chicory protocatechuic acid, caffeic acid, and mix (Figure 5E,F). Scintillation proximity assays further showed that Brussels chicory’s protocatechuic acid or caffeic acid promoted lactate oxidation in C2C12 myotubes, as evidenced by an increase in ^14^CO_2_, an end oxidation product of L-[^14^C(U)] lactate (Figure 5G). This effect seemed to be specific, as Brussels chicory’s protocatechuic acid or caffeic acid did not appreciably affect mitochondrial respiration and glycolysis, as evidenced by comparable levels of cellular OCR and ECAR (Figure 5H,I). These findings suggest that Brussels chicory’s protocatechuic acid, caffeic acid, and the mix promoted lactate oxidation in C2C12 myotubes by targeting LDHB.

### 3.6. Brussels Chicory Phenolic Acids Increased Mitochondria LDHB Without Affecting Its Expression and Then Promoted Lactate Clearance

**Figure 6:**
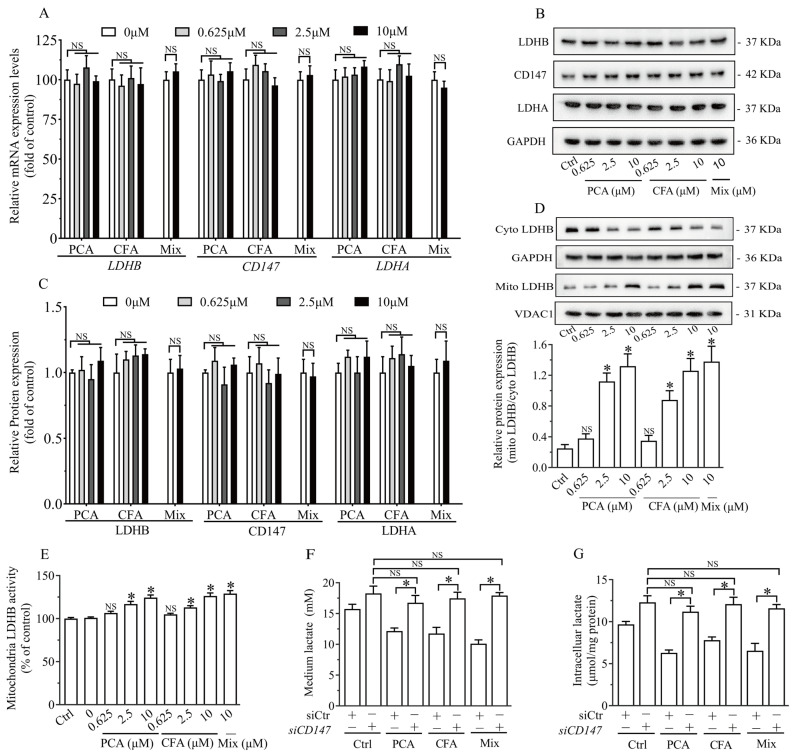
Mitochondria LDHB mediates the effect of Brussels chicory’s phenolic acids on lactate oxidation in the myotubes. qRT-PCR (**A**) and western blot (**B**,**C**) analyses were conducted to assess the key enzymes involved in lactate oxidation and production in C2C12 myotubes treated with PCA, CFA, or a mix for 24 h. The distribution of LDHB between mitochondria and the cytosol was examined in C2C12 myotubes (**D**). The activities of LDHB in mitochondria was measured (**E**). C2C12 myotubes with CD147 knockdown were treated with PCA, CFA, or a mix for 24 h, followed by an additional 6 h incubation with lactate. The levels of intracellular and conditioned medium lactate were then measured (**F**,**G**). Data are mean ± SEM (*n* = 6), Students’ *t* test. D and G, * *p* < 0.05 vs. control. NS, nonsignificant. Abbreviation: LDHB, lactate dehydrogenase B; PCA, protocatechuic acid; CFA, caffeic acid; Mix, containing equal molar concentration of all six Brussels chicory’s phenolic acids; CD147, cluster of differentiation 147; Cyto, cytosol; Mito, mitochondria.

qRT-PCR and western blot analyses showed that the treatment with Brussels chicory’s protocatechuic acid, caffeic acid, and the mix did not affect mRNA or protein expression levels of LDHB and CD147 (a cofactor for LDHB) or LDHA (Figure 6A–C). Interestingly, Brussels chicory’s protocatechuic acid, caffeic acid, and the mix increased the abundance of LDHB protein in mitochondria, while these compounds reduced LDHB protein content in the cytosol (Figure 6D). Consistently, pre-treatment with Brussels chicory’s protocatechuic acid, caffeic acid, and the mix significantly enhanced LDHB enzyme activity in the mitochondria (Figure 6E). Since CD147 is a key cofactor for LDHB function in mitochondrial lactate oxidation, we examined its role in the effect of Brussels chicory’s protocatechuic acid, caffeic acid, and the mix on lactate oxidation. siRNA against CD147 significantly knocked down its protein expression by over 71% (Figure S3). Importantly, the effect of Brussels chicory’s protocatechuic acid, caffeic acid, and the mix on lactate oxidation was not observed in CD147 knockdown in the C2C12 myotubes (Figure 6F,G).

## 4. Discussion

In addition to rigorous training, many recreational and elite athletes still look for an extra edge to improve their exercise performance and post-exercise recovery, often turning to flavonoid-rich fruits (e.g., tart cherry, pomegranate, and blueberry) and vegetables [24,46]. However, as far as we are aware, there are no human exercise studies evaluating the effect of phenolic-acid-rich vegetables on exercise performance and post-exercise recovery. Brussels chicory, a typical Mediterranean leafy vegetable, has, perhaps, the highest content of phenolic acids among vegetables according to the Phenol-Explorer database. Using a short-term, progressive workload, high-intensity, exhaustive aerobic exercise in untrained college students with moderate-intensity physical activity levels, we herein observed two major findings: (i) dietary supplementation with 100 g of Brussels chicory with ~130 mg of phenolic acids for 7 days increases the time to exhaustion by 8.3−12.2% despite exerting no appreciable changes in VO_2_max, enhances blood lactate clearance by 10.6−14.5%, and improves acid–base balance with an increase in bicarbonate in blood circulation of 13.2−16.6%; (ii) physiological achievable doses of Brussels chicory’s phenolic acids are able to enhance lactate oxidation in cultured C2C12 myotubes, an effect reliant on increasing mitochondria LDHB, a limiting enzyme responsible for lactate clearance. In conclusion, our preliminary results support the ergogenic effect of the Brussels chicory on exhaustive aerobic exercise performance and post-exercise recovery, possibly, in part, by promotion of lactate oxidation. The performance of short-term, progressive workload, high-intensity, exhaustive aerobic exercises, such as middle-distance running (800, 1500 m) or swimming (200, 400 m), is largely dependent on the glycolytic system and lactate oxidation. Future work is required to study the potential ergogenic effect of Brussels chicory on different types of exercises, particularly high-intensity exhaustive aerobic exercises [47,48,49]. Given that Brussels chicory contains multiple nutrients and phytochemicals, such as flavonoids (anthocyanin, catechin, quercetin glucuronide) and terpenes (lactucin, lactucopicrin, guaianolide glycosides), it remains unclear whether these compounds also play a role in the ergogenic effect of the Brussels chicory [26,50,51].

The observed changes in exercise performance and post-exercise recovery might be noteworthy. On the one hand, our findings set a proof-of-concept example that phenolic-acid-rich vegetables have a potential ergogenic effect in humans during high-intensity exhaustive aerobic exercise, as most, if not all, previous human exercise studies have studied the role of nitrate-rich beetroot, phenolic-acid-rich coffee, and flavonoid-rich fruits and vegetables (purple cabbage and purple sweet potato leaves) in exercise performance [21,52]. Future works are needed to evaluate the effect of different amounts and timings (before, during, and/or post-exercise) of Brussels chicory consumption on different types of exercise performance. On the other hand, it is generally accepted that approximately 90% of intact flavonoids are not absorbed in the small intestine but, instead, reach the colon, where they are metabolized by gut microbiota into smaller compounds, such as phenolic acids [36,53,54]. Our current finings thus suggest that the ergogenic effect of flavonoid-rich fruits and vegetables might be also linked to these phenolic acid metabolites. Indeed, flavonoid-rich fruits themselves also contain small quantities of phenolic acids; however, phenolic acids’ potential ergogenic effects have been almost, if not completely, ignored in human exercise studies using flavonoid-rich fruits and vegetables.

Although the mechanisms of action by which Brussels chicory consumption increases exercise performance and post-exercise recovery are presently unclear, there are several possible explanations. First, metabolic acidosis is a crucial contributing factor to exercise-induced muscle fatigue during high-intensity exhaustive aerobic exercise [9]. The alkalinizing effect of oral consumption of Brussels chicory, evidenced by an increase in blood HCO_3_^−^ levels immediately after exercise, might partly underpin its benefit on exercise performance during high-intensity treadmill ergometry to exhaustion [8]. Second, a variety of human exercise studies have strongly suggested that the ergogenic activities of flavonoid-rich fruits are partially linked to these well-known antioxidant and anti-inflammatory properties [21]. Because Brussels chicory also contains many phenolic acids, in addition to some flavonoids (e.g., anthocyanin and quercetin) in smaller quantities [21,26], we expect Brussels chicory to improve exercise performance via its antioxidant and anti-inflammatory capacities. To our surprise, our current study showed that this was not the case, as Brussels chicory consumption for 7 days did not alter several blood markers of inflammation (IL-6, IL-8, and CRP) and oxidative stress (activities of SOD, GSH-PX, and CAT). It is, thus, possible that the antioxidant and anti-inflammatory capacities of Brussels chicory may not be linked to its ergogenic effect. Our findings are reminiscent of previous studies in which high doses of vitamins C and E, two popular antioxidant nutrients, fail to enhance, or even hamper, athletic performance [55,56,57]. Third, during high-intensity exercise, the demand for oxygen and energy substrates, as well as metabolic product excretion, is remarkably elevated in exercising muscle fibers [58]. To meet the increased demand, the blood flow to the working musculature is increased. Preclinical animal studies have shown that Brussels chicory and its phenolic acid constitutes could improve the blood flow by promoting endothelial nitric-oxide-mediated vasorelaxation [59,60]. It is, thus, possible that improved blood flow may be one potential mechanism for the Brussels chicory effect on exercise performance and post-exercise recovery, though the status of the blood flow was not analyzed in the current study. However, it should be borne in mind that there was no appreciable change in the maximum oxygen consumption upon Brussels chicory administration, thus allowing us to speculate that blood flow is unlikely to be one action mechanism.

An important finding in the current study is that the alkalinizing effect of oral consumption of Brussels chicory was associated with a reduction in blood lactate, which is in contrast to the notion that an alkalinizing effect typically allows the human muscle to produce more lactate [61,62,63]. This paradox strongly raised the possibility that the alkalinizing effect is secondary to the improved lactate metabolism in muscle fibers. Using cultured C2C12 myotubes, we evaluated the effect of several Brussels chicory’s phenolic acids on lactate metabolism and found that these compounds did increase lactate oxidation via the enrichment of LDHB in mitochondria despite no changes in LDHA-mediated lactate production. Prospectively, if the in vitro findings could extend to in vivo human circumstances, it is logical to hypothesize that the promotion of lactate oxidation is a key mechanism for the alkalinizing effect of Brussels chicory and, in turn, underpins the ergogenic effect observed in the current study. Our findings are reminiscent of previous human exercise studies in which several flavonoid-rich fruits could also improve lactate metabolism during and after high-intensity exhaustive aerobic exercise [64,65,66]. Future work is required to harvest muscle fiber biopsy samples to confirm the effect of Brussels chicory on mitochondrial LDHB-mediated lactate oxidation. Indeed, genetic studies on mice have shown that, during high-intensity aerobic treadmill running tests to exhaustion, LDHB overexpression reduces blood lactate levels and enhances exercise performance [67] and vice versa [17].

It should be pointed out that, in addition to Brussels chicory, chicory is cultivated worldwide as several other edible varieties, such as radicchio (also known as the Italian chicory), curly endive, treviso, and tardivo endive. These different varieties have comparable phenolic acid profiles and content to Brussels chicory [68]. Moreover, the leafy vegetable lettuce (*Lactuca sativa* L.), more popular than chicory, also belongs to the family of Asteraceae and has an abundant content of phenolic acids [69]. Therefore, we are wondering whether lettuce has a similar ergogenic effect in humans to Brussels chicory. This question is worthy of further study, as it would greatly facilitate consumer acceptability and inclusion in regular diets, potentially increasing the translational potential of this research.

This study has several limitations. One obvious limitation is the relatively small sample size, which may limit the generalizability of the findings. Small sample sizes can reduce the statistical power of the study, potentially leading to inconclusive or biased results. The sample in this study was limited to untrained college students, which may not fully represent the wider population. Therefore, caution should be exercised when attempting to generalize these results to other groups, such as individuals with different fitness levels, age or gender groups, or health conditions. Further, the study focused on short-term interventions, limiting the ability to draw conclusions about the long-term effects of Brussels chicory supplementation on exercise performance and post-exercise recovery. While acute responses are important, the cumulative effects of sustained dietary changes over time could differ. A longer duration of supplementation may be necessary to capture potential adaptations that could influence both performance and recovery. 

## 5. Conclusions

In conclusion, to our knowledge, this is the first pilot study to provide direct evidence of the impact of Brussels chicory on exercise performance and post-exercise recovery. Brussels chicory is able to increase time to exhaustion during high-intensity aerobic exercise and also promotes post-exercise recovery, both of which are, possibly, in part reliant on facilitating LDHB-mediated lactate clearance in the muscles. Given that high-intensity, short-term aerobic exercise tests to exhaustion typically have a high intraparticipant variation in performance of approximately 5% to 10% [70,71], close to the Brussels-chicory-elicited performance improvement (8.3% for men and 12.2% for women), further studies to confirm the ergogenic effect of Brussels chicory with different modalities, intensities, and durations of exercise are required. Nevertheless, our current findings support the notion that Brussels chicory might be a promising ergogenic agent in untrained humans during short-term, progressive workload, high-intensity, exhaustive aerobic exercise.

## Figures and Tables

**Table 1:** Bruce protocol.

Stages	Speed Mph (km/h)	Grade (%)	Duration (min)
Warm-up	1.7 (2.7)	0	3
Warm-up	1.7 (2.7)	5	3
1	1.7 (2.7)	10	3
2	2.5 (4.0)	12	3
3	3.4 (5.4)	14	3
4	4.2 (6.7)	16	3
5	5.0 (8.0)	18	3
6	5.5 (8.8)	20	3
7	6.0 (9.6)	22	3

**Table 2:** Baseline characteristics of the healthy college students in the chicory juice and placebo groups ^a^.

Characteristics	Placebo Then Brussels Chicory (*n* = 16)	Brussels Chicory Then Placebo (*n* = 16)	*p* ^b^
Age (years)	24.31 ± 3.52	22.37 ± 2.22	0.097
Gender			0.432
Women [*n* (%)]	7 (44.75%)	7 (44.75%)	
Men [*n* (%)]	9 (56.25%)	9 (56.25%)	
Weight (kg)	62.95 ± 6.13	65.63 ± 6.85	0.056
Body mass index (kg/m^2^)	21.60 ± 1.78	22.67 ± 1.54	0.213
Percentage body fat (%)	17.23 ± 4.62	20.44 ± 5.83	0.069
Systolic blood pressure (mmHg)	114.95 ± 5.51	118.05 ± 6.96	0.369
Diastolic blood pressure (mmHg)	72.85 ± 4.97	76.72 ± 5.54	0.082
Heart rate (beats/min)	73.15 ± 6.03	72.43 ± 8.15	0.724
Physical activity level (MET-min/w)	1802.27 ± 494.45	2132.12 ± 505.77	0.432
Walking (MET-min/w)	808.09 (724.10, 995.17)	802.45 (620.46, 953.57)	0.386
MPA ^c^ (MET-min/w)	801.07 (526.18, 890.67)	581.21 (451.82, 702.17)	0.142
VPA ^d^ (MET-min/w)	458.42 (283.43, 640.83)	326.90 (217.93, 541.82)	0.152
Energy (kcal/day)	1963.32 ± 266.55	2047.86 ± 262.91	0.102
Carbohydrates (g/day)	312.67 ± 57.82	349.45 ± 56.93	0.200
Proteins (g/day)	64.33 ± 11.58	69.28 ± 10.49	0.445
Fat (g/day)	48.64 ± 11.33	54.86 ± 12.44	0.521

^a^ Data are expressed as the mean ± SD or median (IQR) for continuous variables and n (%) for categorical variables. ^b^ Independent Student’s *t*-test, Mann–Whitney U, and chi-square tests were conducted to examine the differences in baseline characteristics. ^c^ MPA: moderate physical activity. ^d^ VPA: vigorous physical activity.

**Table 3:** Comparison of blood parameters 0 and 60 min post-exhaustive aerobic exercise in men based on intervention ^a^.

	0 min Post-Exercise		60 min Post-Exercise		*p* ^c^	*p* ^d^	*p* ^e^
	Placebo	Chicory ^b^	Placebo	Chicory			
IL-6 pg/mL	2.84 ± 1.43	2.68 ± 1.24	3.08 ± 1.36	2.82 ± 1.54	0.424	0.623	0.829
IL-8 pg/mL	3.21 ± 1.23	2.91 ± 1.52	3.30 ± 1.98	3.09 ± 2.11	0.459	0.684	0.878
CRP mg/dL	0.75 ± 0.43	0.63 ± 0.38	0.92 ± 0.65	0.77 ± 0.45	0.389	0.054	0.852
SOD activity mU/mg prot	0.43 ± 0.16	0.56 ± 0.20	0.41 ± 0.19	0.49 ± 0.28	0.052	0.266	0.428
GSH-PX activity mU/mg prot	8.84 ± 3.43	9.68 ± 3.24	9.08 ± 4.36	10.82 ± 4.54	0.171	0.445	0.583
CAT activity mU/mg prot	44.51 ± 17.08	48.48 ± 21.44	46.57 ± 21.07	53.56 ± 24.06	0.170	0.525	0.715

^a^ Data are expressed as the mean ± SD. Results from two-way repeated measures ANOVA tests. ^b^ Brussels chicory. ^c^ *p*-value for intervention (placebo and Brussels chicory). ^d^ *p*-value for time (0 and 60 min). ^e^ *p*-value for intervention × time. Abbreviation: IL, Interleukin; CRP, C-reactive protein; SOD, superoxide dismutase; GSH-PX, glutathione peroxidase; CAT, catalase; prot, protein; ANOVA, analysis of variance.

**Table 4:** Comparison of blood parameters 0 and 60 min post-exhaustive aerobic exercise in women based on intervention ^a^.

	0 min Post-Exercise		60 min Post-Exercise		*p* ^c^	*p* ^d^	*p* ^e^
	Placebo	Chicory ^b^	Placebo	Chicory			
IL-6 pg/mL	2.44 ± 1.28	1.93 ± 1.23	2.01 ± 1.20	1.94 ± 1.72	0.352	0.630	0.538
IL-8 pg/mL	3.10 ± 1.65	2.26 ± 1.77	3.23 ± 1.67	2.55 ± 1.22	0.055	0.713	0.841
CRP mg/dL	1.04 ± 0.65	0.85 ± 0.16	1.16 ± 0.70	1.18 ± 0.63	0.439	0.266	0.252
SOD activity mU/mg prot	0.49 ± 0.29	0.63 ± 0.33	0.44 ± 0.25	0.58 ± 0.18	0.051	0.989	0.437
GSH-PX activity mU/mg prot	10.44 ± 4.28	8.93 ± 3.23	9.61 ± 2.20	9.94 ± 3.72	0.270	0.941	0.187
CAT activity mU/mg prot	56.47 ± 26.09	50.44 ± 19.13	52.49 ± 19.05	55.51 ± 21.08	0.595	0.942	0.211

^a^ Data are expressed as the mean ± SD. Results from two-way repeated measures ANOVA tests. ^b^ Brussels chicory. ^c^ *p*-value for intervention (placebo and Brussels chicory). ^d^ *p*-value for time (0 and 60 min). ^e^ *p*-value for intervention × time. Abbreviation: IL, Interleukin; CRP, C-reactive protein; SOD, superoxide dismutase; GSH-PX, glutathione peroxidase; CAT, catalase; prot, protein; ANOVA, analysis of variance.

## Data Availability

All data that support the findings of this study are available from the corresponding author upon reasonable request.

## Supplementary Materials

The following supporting information can be downloaded at: https://www.mdpi.com/article/10.3390/nu17020365/s1. Figure S1: Cell viability of C2C12 myotubes incubated with Mix or its vehicle DMSO for 24 h was assessed by MTT assay (A) and LDH leakage assay (B). Figure S2: C2C12 myotubes were untreated, or transfected with control siRNA(siCtr) or siRNA directed against LDHB (siLDHB) with 20 nM for 24 h. Figure S3: C2C12 myotubes were untreated, or transfected with control siRNA(siCtr) or siRNA directed against CD147 (siCD147) with 20 nM for 24 h.

## Abbreviations

ANOVA, analysis of variance; AUC, area under the curve; BMI, body mass index; CAT, catalase; CFA, caffeic acid; CRA, chlorogenic acid; CTA, caftaric acid; CD147, cluster of differentiation 147; CRP, C-reaction protein; DBP, diastolic blood pressure; ECAR, extracellular acidification rate; GA, gallic acid; GSH-PX, glutathione peroxidase; HCO_3_^−^, bicarbonate; IL-6, interleukin-6; IL-8, interleukin-8; LDH, lactate dehydrogenase; MTT, methyl thiazolyl tetrazolium; OCR, oxygen consumption rate; PCA, protocatechuic acid; PHBA, p-hydroxybenzoic acid; qRT-PCR, quantitative real-time PCR; SBP, systolic blood pressure; SOD, superoxide dismutase; VO_2_max, maximal oxygen consumption.
