# Supplementary material for: Brussels Chicory Enhances Exhaustive Aerobic Exercise Performance and Post-Exercise Recovery, Possibly Through Promotion of Lactate Oxidation: A Pilot Randomized, Single-Blind, Placebo-Controlled, Two-Way Crossover Study
Source: Nutrients. 2025 Jan 20;17(2):365. doi: 10.3390/nu17020365 (PMC11769108; doi:10.3390/nu17020365)
Supplement: Supplementary file 1 [file nutrients-17-00365-s001.zip › nutrients-3430270-supplementary.pdf]

Brussels chicory enhances exhaustive aerobic exercise performance and post-exercise recovery possibly through promotion of lactate oxidation: a pilot randomized, single-blind, placebo-controlled, 2-way crossover study

Yihui Mao [1†], Junhao Huang [2†], Shuangshuang Li [1], Guanyu Chen [1], Yushi Du [1], Mengxi Kang [1], Shasha Zhu [1], Wenyu Zhang [1], Qiuhui Xu [1], Yihan Wang [1], Wenhua Ling [1, 4], Xijuan Luo [3*], Dongliang Wang [1, 4*]

[1] Department of Nutrition, School of Public Health, Sun Yat-sen University (Northern Campus), Guangzhou, People' s Republic of China. (Yihui Mao, maoyh23@mail2.sysu.edu.cn; Shuangshuang Li, lishsh86@mail2.sysu.edu.cn; Guanyu Chen, chengy268@mail2.sysu.edu.cn; Yushi Du, duysh5@mail2.sysu.edu.cn; Mengxi Kang, kangmx@mail2.sysu.edu.cn; Shasha Zhu, zhushsh8@mail2.sysu.edu.cn; Wenyu Zhang, zhangwy69@mail2.sysu.edu.cn; Qiuhui Xu, xuqh36@mail2.sysu.edu.cn; Yihan Wang, wangyh266@mail2.sysu.edu.cn); Wenhua Ling, lingwh@mail.sysu.edu.cn.

[2] Guangdong Provincial Key Laboratory of Sports and Health Promotion, Scientific Research Center, Guangzhou Sport University, Guangzhou, People' s Republic of China; Junhao Huang, junhaohuang2006@hotmail.com

[3] Department of Sports, Sun Yat-sen University, Guangzhou, People' s Republic of China.

[4] Guangdong Provincial Key Laboratory for Food, Nutrition and Health, Guangzhou, People' s Republic of China.

[†] Yihui Mao and Junhao Huang contributed equally to this work.

[*] Dongliang Wang and Xijuan Luo contributed equally as corresponding authors.

[*] **Corresponding Author**

Prof. Dongliang Wang

Department of Nutrition, School of Public Health, Sun Yat-sen University (Northern Campus),

74 Zhongshan Road II, Guangzhou 510080, People' s Republic of China.

Tel: 86-20-87332472

Fax: 86-20-87330446

E-mail: wdliang@mail.sysu.edu.cn

Assoc. Prof. Xijuan Luo

Department of Sports, Sun Yat-sen University, 135 West Xingang Road, Guangzhou 510275,

People' s Republic of China.

E-mail: luoxijuan@mail.sysu.edu.cn

Figure

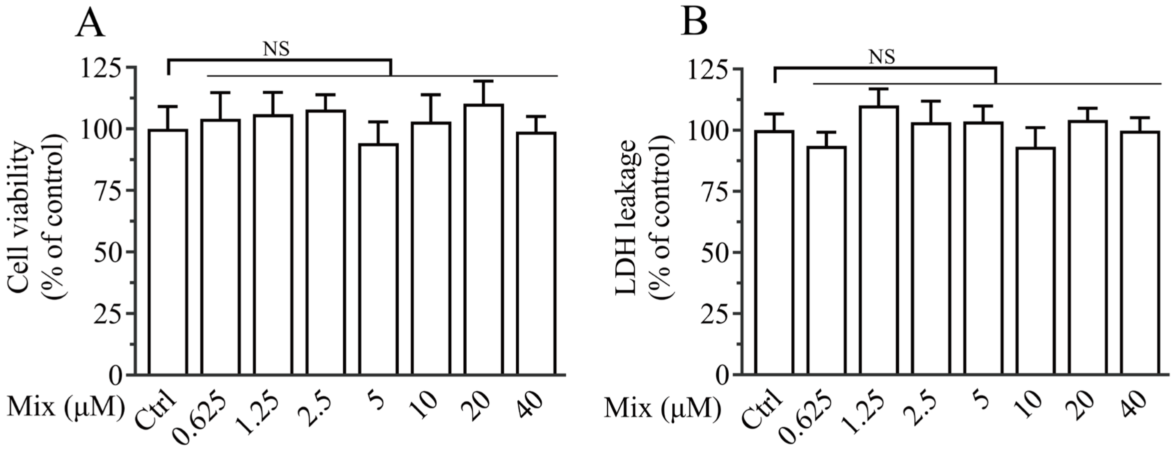

Supporting Information Figure S1 Cell viability of C2C12 myotubes incubated with Mix or its vehicle DMSO for 24 hours was assessed by MTT assay (A) and LDH leakage assay (B). Data were Mean ± SEM (n = 6), Student's t test. NS, nonsignificant. Abbreviation: Mix, containing equal molar concentration of all 6 Brussels chicory phenolic acids including protocatechuic acid, gallic acid, p-Hydroxybenzoic acid, chlorogenic acid, caftaric acid and caffeic acid.

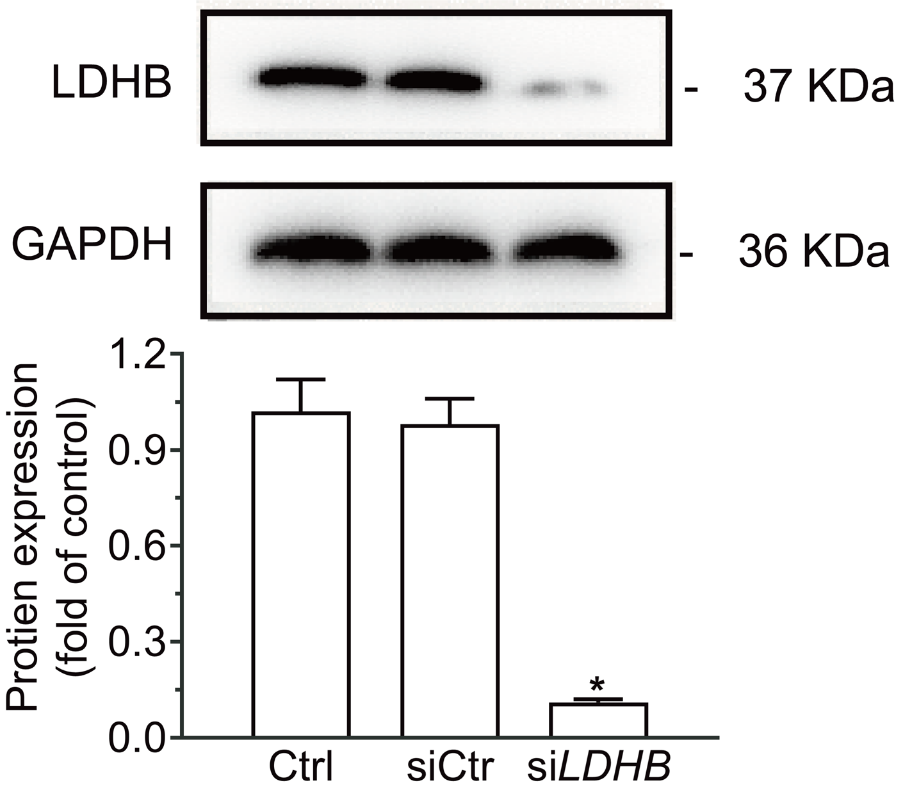

Supporting Information Figure S2 C2C12 myotubes were untreated, or transfected with control siRNA(siCtr) or siRNA directed against LDHB (siLDHB) with 20 nM for 24 hours. LDHB knockdown efficiency was then determined by Western blot. Data were Mean ± SEM (n=6), Student's t test, *P<0.05 vs siCtr. Abbreviation: LDHB, lactate dehydrogenase B.

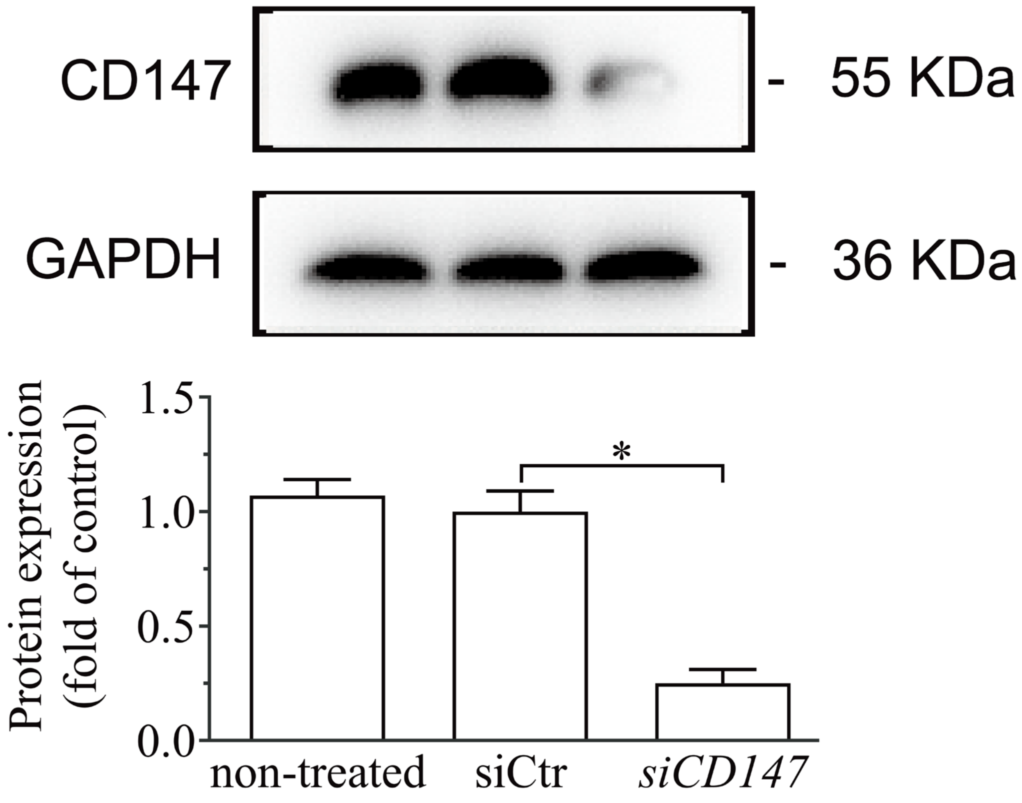

Supporting Information Figure S3 C2C12 myotubes were untreated, or transfected with control siRNA(siCtr) or siRNA directed against CD147 (siCD147) with 20 nM for 24 hours. CD147 knockdown efficiency was then determined by Western blot. Data were Mean ± SEM (n = 6), Student's t test, *P<0.05 vs siCtr. Abbreviation: CD147, cluster of differentiation 147.
